# *Bluephage*, a method for efficient detection of somatic coliphages in one hundred milliliter water samples

**DOI:** 10.1038/s41598-020-60071-w

**Published:** 2020-02-19

**Authors:** Javier Méndez, Daniel Toribio-Avedillo, Raquel Mangas-Casas, Judit Martínez-González

**Affiliations:** Department of Genetics, Microbiology and Statistics. Faculty of Biology, University of Barcelona, Av. Diagonal 643, 08028 Barcelona, Spain

**Keywords:** Applied microbiology, Bacteriophages

## Abstract

Emerging water quality guidelines and regulations require the absence of somatic coliphages in 100 mL of water, yet the efficiency of standardized methods to test this volume of sample is questionable. A recently described procedure, Bluephage, using a modified *E. coli* host strain, overcomes some of the methodological limitations of standardized methods. In a maximum of 6.5 hours (2.5 hours for pre-growing the host strain and 4 hours for the presence/absence test), Bluephage allows the direct detection of one plaque-forming unit (PFU) in a 100 mL water sample. The test shows high levels of specificity for somatic coliphages and comparable accuracy with standardized methods.

## Introduction

Coliphages, viruses infecting *Escherichia coli*, have been included or are being considered for inclusion in water quality regulations and guidelines around the world^1–6^. Some of these regulations, such as those for different types of drinking water^5,6^, prescribe or recommend the absence of somatic coliphages in 100 mL water samples.

Widely accepted standardized methods for determining somatic coliphages are available^7–9^. The host strains and media endorsed in the ISO (International Organization for Standardization) standard 10705-2:2000^7^ and the USEPA (U.S. Environmental Protection Agency) 1601 and 1602 methods^8,9^ provide similar results^10–12^. To determine coliphage absence in 100 mL water samples, which requires analysis of the full 100 mL volume, the following standardized procedures are currently used: (1) the presence/ absence test according to ISO or USEPA; (2) the double agar layer (DAL) assay as indicated in ISO^7^, which requires the concentration of phages in 100 mL of water sample followed by phage enumeration in the full concentrate, and (3) the USEPA’s single agar layer (SAL) assay^8^, which is applied to 10 replicas of 10 mL. According to ISO and USEPA standards, the presence/absence test requires 2 steps (enrichment and spot test) and needs more than one working day to obtain results. On the other hand, both the concentration^13,14^ and SAL procedures are complex and suffer from loss of efficiency in coliphage recovery^10,15^. Feasible, efficient, fast and user-friendly methods to determine the presence of coliphages in 100 mL water samples are therefore required. Recent progress in this field^16–18^ includes the development of Bluephage method^18^, whose application can facilitate the routine implementation of somatic coliphage determination in laboratories with minimal equipment and expertise requirements.

To detect phage concentrations as low as one in a given volume is challenging, as viral particles have an uneven spatial distribution in watery suspensions, theoretically following the Poisson model^19^. Accordingly, to assess the accuracy and detection limit of different phage determination methods, a statistical approach was used to calculate the distribution in aliquots of suspension and consequently the expected percentage of positive samples in a set of recipients prepared to contain about one phage per 100 mL.

The aim of this study was to compare the accuracy of the Bluephage method in testing for the presence/absence of somatic coliphages in 100 mL volumes of water, as well as to assess its specificity and detection limit compared to the reference method ISO-DAL.

## Materials and Methods

### Bacteriophage enumeration

The ISO DAL standardized method^7^ was used to count somatic coliphages.

### Susceptibility of host strains GW5 and CB10 to different coliphages

The sensitivity of both host strains to coliphage isolates was tested with pure cultures of phages of our lab library, some of them coming from culture collections and others isolated by us and characterized by electron microscopy^20^. The spot test performed according to ISO^7^ was carried out with bacteriophages belonging to different families of somatic bacteriophages: *Microviridae* (Phi X174 and M4), *Myoviridae* (SOM 1, SOM 3, SOM 5, SOM 8, SOM 15, SCH2, SCH10 and STER 5), *Podoviridae* (933 W), *Siphoviridae* (SOM 4, SOM7, SOM 28, SSAU 9, MAR 1 and SCH4) and *Tectiviridae* (PDR1). Moreover, F-specific bacteriophages were tested: *Inoviridae* (F1) and *Leviviridae* (MS2, GA and Qβ).

As well, the cross-reaction with phages partially purified from well-isolated and visually different plaques on the two strain by was tested. Plaques were obtained applying the ISO DAL method to a variety of fecally polluted water samples. Thus, 30 plaques obtained on WG5 and 30 on CB10, were purified and their capability to infect was tested by the spot test in both WG5 and CB10 strains.

### Reference coliphage suspensions

Reference suspensions of naturally occurring somatic coliphages were prepared according to Méndez *et al*.^21^. Briefly, influent raw sewage from a wastewater treatment plant was centrifuged for 10 min at 2000 × *g*, and the supernatant was filtered through 0.22 µm low protein-binding polyethersulfone membrane filters. The naturally occurring somatic coliphages in the resulting suspensions were quantified and after dilution concentrations ranging from 50 to 100 PFU per mL were obtained. Glycerol was added to give a final concentration of 5 or 10% (v/v). The suspensions were distributed into vials of 1.5 or 2.5 mL and stored at (−70 ± 10) °C.

### Quality control of the reference suspension

**Figure 1:**
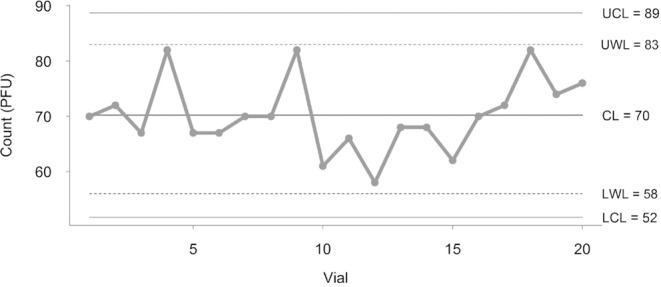
Control chart of the reference material used for the comparison test. UCL, UWL, CL, LWL and LCL, refer to, respectively, the upper control limit (+3 sigma), the upper warning limit (+2 sigma); the central line (mean value), the lower warning limit (-2 sigma) and LCL lower control limit (-3 sigma).

The quality and stability of the reference phage suspension was monitored by a control chart prepared in accordance with van Dommelen^22^. For this purpose, 20 vials of each batch were enumerated on different days. Based on the results, the mean ($\bar{x}$) and sigma (s) were calculated and then the warning limits ($\bar{x}\pm 2s$) and control limits ($\bar{x}\pm 3s$). In each successive experiment, one vial of the batch was analyzed, and the result was included in the chart. Results were considered out of control in the case of a single violation of the control limit ($\bar{x}\pm 3s$) and if two out of three observations in a row exceeded the same warning limit ($\bar{x}\pm 2s$). Figure 1 shows the control chart of the phage suspension used.

### Presence/absence in 100 ml of water by Bluephage

Briefly, an aliquot of a freeze-dried culture of *E. coli* strain CB10^18^, containing about 10^9^ cells, was suspended in 10 mL of Modified Scholtens’ Broth (MSB)^7^. The mixture was incubated at (36 ± 1) °C for 2.5 hours with gentle shaking. When ready, 10 mL of the culture was added to a 100 mL volume of water for testing. The culture was previously obtained using a dry culture medium (fast kit BPF-SPA, Bluephage S. L., Barcelona) containing the necessary compounds to obtain the exact broth composition as described by Muniesa *et al*.^18^. The water sample receiving the CB10 culture was swirled gently until thoroughly mixed and incubated at (36 ± 1) °C. The test results were read after 3.5 hours and the incubation was never extended more than 4 hours from the beginning.

### Detection performance

Detection was performed over a period of several weeks and by two different operators, who each carried out a similar number of tests. The control chart of the reference suspension was used throughout this period, in accordance with the quality control schemes followed, and there were no deviations in the counts. Aliquoted volumes of 10 µL, 25 µL, 75 µL and 100 μL of the reference suspension were titrated by the ISO DAL assay^7^ and equal quantities of the reference suspension were added to containers containing 100 mL of commercial mineral water, and submitted to a presence-absence test with Bluephage, as described above.

As negative control for Bluephage method, 80 commercial mineral water samples were analyzed.

### Statistical analysis

The Kolmogorov-Smirnov test (KS), the Akaike information criteria (AIC), the test of equal or given proportions and Wilcoxon test were carried out under R, version 3.5.3^23^. Distribution fitting was done using the library fitdistrplus, version 1.0-14^24^. Statistical bootstrapping was completed using the library boot, version 1.3-20^25,26^. In addition, the most probable number (MPN) of coliphage concentrations in the bacteriophage suspensions without 100% positive detection frequency was calculated using the library MPN, version 0.3.0^27^, calculating the confidence intervals using the Jarvis approach^28^.

## Results and Discussion

Both the host strains and the method are specific for those somatic coliphages that are also detected by the strain WG5 and the ISO DAL method.

Strain CB10 was, as it is strain WG5, susceptible of being infected by 18 bacteriophages belonging to five families of somatic coliphages, four of them being the most frequently observed in faecally contaminated waters^29^. As well, both strains were sensitive to 60 recent bacteriophage isolates, and thus strain CB10 follows being susceptible to phages infecting WG5 and all phages isolated in CB10 infect strain WG5. In contrast, neither strain WG5 nor strain CB10 were susceptible to the F-specific bacteriophages tested. Consequently, it can be deduced that there is not change in phage susceptibility of strain CB10 with respect to strain W5.

None of the 80 negative controls changed to blue in 4 hours, indicating an absence of phages. The Bluephage method therefore showed a very good specificity, showing no false positive results in 80 samples, or according with the Poisson distribution, with lesser than 1.25% of false positives.

Thus, it can be concluded that both the host strains and the method are specific for those somatic coliphages that are also detected by the strain WG5 and the ISO DAL method.

The phage concentration values obtained by the different methods are displayed in Table 1. The bacteriophages counted by the ISO DAL assay in 10, 25 and 75 μL volumes of the reference suspension as well as the theoretical numbers calculated from the control chart of the reference suspension both fitted a Poison distribution better than a binomial or a normal distribution (Table 2). The AIC were applied as selection criteria, and the KS test was carried out to verify that the real counts and calculated probability distribution functions did not differ statistically. Accordingly, the Poison distribution of the theoretical values in 10 µL, 25 µL and 75 µL of the reference suspension allowed the prediction of the number of positive detections in these three volumes, which was compared with the number of positive detections obtained with the ISO DAL and Bluephage methods (Table 3).

**Table 1:** Results after enumeration using the ISO DAL method and presence/absence with the % of positive enrichments using the Bluephage method.

Method							
Control Chart			ISO-DAL			Bluephage	
Vol. tested	n	Central line (control limits)^a^	Mean and (95% C.I.)^b^	MPN and (95% C.I.)^c^	% pos.	% pos.	MPN and (95% C.I.)^c^
Method							
0 μl	80	0	0	0 (0–0.037)	0	0	0 (0–0.037)
10 μl	42	0.70 (0.52–0.89)	0.45 (0.26–0.62)	0.51 (0.32–0.84)	40.4	38.1	0.48 (0.29–0.79)
25 μl	18	1.80 (1.30–2.23)	1.78 (0.94–2.50)	1.28 (0.72–2.29)	72.2	72.2	1.28 (0.72–2.29)
75 μl	10	5.3 (3.90–6.69)	5.60 (4.30–6.90)	Non computable	100	100	Non computable
100 μl	10	7.00 (5.20–8.92)	10.10 (7.50–12.10)	Non computable	100	100	Non computable

^a^Theoretical values of the reference material according to the inoculated volume, theoretical mean and control limits; ^b^mean and confidence intervals obtained by statistical bootstrapping; ^c^MPN and confidence interval calculated according to Jarvis^26^.

**Table 2:** Summary of the parameters of the preferred probability distribution functions for each volume of the reference materials titrated by the ISO DAL assay. *Chi-squared *p*-value.

Vol. tested	AIC			Distribution	*p-*value*	λ	C.I. of λ	
	Poisson	Neg. Binomial	Normal				2.5%	97.5%
10 μl	72.92	74.92	78.25	Poisson	0.261	0.452	0.286	0.643
25 μl	67.18	67.49	72.40	Poisson	0.427	1.778	1.056	2.611
75 μl	46.10	48.10	48.15	Poisson	0.155	5.600	4.100	7.000

**Table-3:** Comparison between the number of positive samples assuming the Poisson probability distribution functions.

Vol. tested	n	Positive samples (%)			Wil. test^§^
		Theoretical	ISO	Bluephage	*p-value*
10 μl	42	36.59	40.40	38.10	>0.05
25 μl	18	83.20	72.22	72.22	>0.05
75 μl	10	99.65%	100.00	100.00	*

^§^Wilcoxon test results (both comparisons); * non-computable.

The rate of positive detections using Bluephage method was identical (p > 0.05, test of equal or given proportions) to that of the theoretical test and the ISO DAL assay.

The content of all containers receiving 75 µL and 100 μL of the reference phage suspension turned blue after 4 hours, indicating the presence of phages, and the ISO DAL assay gave always-positive counts. Therefore, the results corresponding to tests where 100 μL of the reference suspension was added to 100 mL were not included in the determination of accuracy and detection limit.

The containers receiving 10 µL and 25 μL of the reference suspension showed different percentages of positive/negative detections as it is shown in Table 1. In addition, zero-values were detected in a number of tests by the ISO DAL assay carried out in parallel. From the results of the Bluephage method (positive/negative) for each inoculated volume, it was possible to determine the mean value expressed as MPN of bacteriophages in 100 mL of the inoculated samples (Table 2). These values fit with the theoretical numbers added to each container according to the phage concentration of the reference suspension and the counts obtained using the ISO DAL assay.

Moreover, using the Poisson distributions derived from the percentage of positive samples (Table 3) inoculated with 10 µL and 25 µL a bootstrapping simulation for the Bluephage method was carried out to calculate the accuracy and specificity of this method. Briefly, the first step for it was determining the λ-parameters, which were estimated assuming the percentage of negative samples as the probability of 0-counts. Then, 10,000 runs were simulated for the 42 counts inoculating 10 µL and for the 18 counts inoculating 25 µ. Counts containing ≥1 phage were considered positive. The simulated mean values of positive and negative samples were compared to the real values obtained with ISO-DAL in order to calculate the accuracy and the specifity of the method. After the simulation the mean accuracy value was 96.67% with a confidence interval of 92.67 to 99.33%; as well, the mean specificity value was 97.78% with a confidence interval ranging from 94.3 to 100%, which fits with the showed results with the sensitivity test performed.

According to these results, the Bluephage method was as accurate as the ISO DAL assay for somatic coliphage detection in 100 mL water samples.

The limit of detection was calculated from the proportions of 10µL-inoculated samples, titrated following the ISO DAL procedure, that contained 1 bacteriophage, versus the proportion of positive samples (≥1 bacteriophage) detected using Bluephage method. The percentage of positive samples for the ISO DAL and Bluephage methods were 35.71% and 38.01%, respectively (Table 3). As the Poisson distribution describes the probability of *n* number of events occurring in an interval given an average rate (λ), when *n* = 0, the probability function can be described as e^-λ^. In this particular case, the estimated λ-values for samples titrated according to the ISO DAL method and tested by Bluephage method were 0.4418 and 0.4796, respectively. To check for any statistical difference between both values, a bootstrapping analysis was carried out to determine the interval of λ-values, which did not statistically differ from the λ-value of ISO DAL when the KS test was applied. The interval of λ-values ranged from 0.4032 to 0.4826, indicating that the performance of Bluephage method applied to samples containing low bacteriophage concentrations did not differ from the ISO DAL titration.

Summarizing, it can be concluded that the Bluephage method is a specific, accurate and a rapid presence/absence feasible method to detect somatic coliphages, with a detection limit of 1 PFU per 100 mL, applicable to 100 mL water samples.
